# Provider reported challenges with completing death certificates: A focus group study demonstrating potential sources of error

**DOI:** 10.1371/journal.pone.0268566

**Published:** 2022-05-20

**Authors:** Allie Morgan, Thomas Andrew, Sylvia M. A. Guerra, Valeria Luna, Louise Davies, Judy R. Rees

**Affiliations:** 1 Geisel School of Medicine, Dartmouth College, Hanover, NH, United States of America; 2 Office of Chief Medical Examiner, Hanover, State of New Hampshire, United States of America; 3 New Hampshire State Cancer Registry, Hanover, New Hampshire, United States of America; 4 The Dartmouth Institute for Health Policy and Clinical Practice, Hanover, NH, United States of America; 5 The VA Outcomes Group, Department of Veterans Affairs Medical Center, White River Junction, VT, United States of America; 6 Norris Cotton Cancer Center, Lebanon, NH, United States of America; Massachusetts General Hospital, UNITED STATES

## Abstract

**Objectives:**

To characterize the experiences of providers in completing the cause of death section on death certificates, with particular reference to deaths in people who have cancer.

**Methods:**

Focus groups were conducted until thematic saturation was reached, resulting in four groups over three months. Participants were from a variety of specialties and levels and types of training. Focus groups were recorded and transcribed verbatim and analyzed using constant comparison analysis.

**Results:**

Three types of challenges to case classification were identified. 1) Infrastructural and procedural challenges encountered when completing death certificates, including the rigid structure of the form, lack of training in its completion, and lack of real-time feedback. 2) Clinical uncertainty and the varied approaches providers take to determine the cause of death based on their perception of the purpose of the death certificate. 3) Choosing cause of death in decedents with a history of cancer.

**Conclusions:**

There are specific and substantial challenges in the death certification process that lead to errors in documenting the cause of death, but many of these challenges could be addressed with structural change to the forms or mechanism of training. Using these data to inform change could improve the death certification process and reliability of this data.

## Introduction

Death certificates have been used to track public health data in the United States since 1844 when Massachusetts passed the first law requiring cause of death to be reported to the state [1]. Since then, death certificates have become the basis for mortality statistics and directly influence medical practice, research and public health policy [2]. Taken individually, these forms can provide closure for next of kin and assist in estate and insurance settlements. When taken collectively, death certificates describe the health of the nation, reveal public health threats and progress, and demonstrate disparities between communities or populations [3–10].

Despite their crucial importance, numerous studies have demonstrated high rates of error in death certificates, especially in the cause of death section [11–19]. For example, a 2017 study in Vermont found that 51% of death certificates had a major error that impacted the interpretation of the primary cause or contributing causes of death [20]. In a 2010 study, deaths from cardiovascular disease in New York City were overestimated by 51% in adults ages 35–74 [21]. These errors have significant impacts on the public health data that underlie disease specific mortality rates, track health disparities and inform research and policy priorities. For example, in a study by Yin et al, misclassification of colon and rectal cancer deaths on the death certificate led to an inflation of the 5-year cause-specific survival rate for rectal cancer from 64.9% to 81.2% [22]. Despite ample evidence of errors in death certificates in the United States [11–19], and evidence that these errors are increasing in frequency [23], it is not clear what is causing these errors or how to prevent them. This study aims to better understand the challenges faced by providers when they complete the cause of death section of death certificates, how those challenges might contribute to sources of error, and any specific challenges relating to cancer-related deaths.

## Methods

In the Spring of 2019 four in-person focus groups were conducted with 2–5 participants in each group. Focus groups were chosen for this study to replicate the discussions that may occur between providers when faced with challenges in death certification, and to investigate the degree of consensus between providers on the challenges involved in determining the cause of death. After the fourth focus group, thematic saturation was reached, a finding in line with Guest et. al who found that 80% of themes are captured in 2–3 focus groups and 90% are captured in 3–6 focus groups [24]. Purposive sampling [25] was used to recruit providers from a variety of specialties, levels of training and type of practice, using group email invitations. The study was conducted at a single academic institution in rural New England. A moderator (AM) oversaw each group and two additional researchers (SG and VL) took notes on participant tone and body language. The moderator was a medical student with training in qualitative research methods. Her role was to provide the group with standardized discussion questions and ask follow-up questions on new ideas presented, while managing the time and ensuring all focus group members had the chance to speak. Group bias was minimized by creating groups where providers did not know one another and by masking the provider type to avoid the potential of presumed hierarchy influencing provider responses. The 60–90 minute groups were conducted in private hospital conference rooms with participants seated at a single table to facilitate discussion. The focus groups were recorded with an audio recording device and then transcribed verbatim for analysis by the study team. The COREQ checklist was followed to present the study findings [26].

Each group was asked to consider eight questions and one case study adapted from publicly available training materials to reflect a situation of clinical uncertainty (S1 and S2 Appendices). Each participant was asked to complete the cause of death section of a blank U.S Standard Certificate of Death for the patient or to describe what approach they would take if they were uncertain. This was then used to start a discussion about what specific challenges the providers faced as they completed the death certificate.

Providers were offered a $30 Amazon gift card and a free meal during the focus group. IRB approval was granted by the Dartmouth College Committee for the Protection of Human Subjects. We were granted IRB exemption for this study, IRB number: STUDY00031374. Written informed consent was obtained from all participants prior to their participation in the focus groups. No minors were included in this study.

Focus group transcripts were analyzed using constant comparison analysis and QDA Miner software, a research method based in grounded theory that groups data into units then categories and finally a larger theme [25]. The iterative process of constant comparison analysis allows for identification of broad themes and patterns that can be used to generate theories, an approach that is particularly useful in qualitative studies aimed at exploring new ideas. AM analyzed the data with review during the process together with JR, any discrepancies in interpretation were discussed with the research team and resolved by consensus. Representative quotations for each area of challenge were selected from the transcripts to provide examples of statements made by participants. The causes of death chosen by participants in the case study were summarized.

## Results

45 providers were invited, 25 providers responded, and 14 participated in one of four scheduled focus groups. Every focus group included both in-patient and out-patient providers and both men and women. Internal medicine was the most commonly represented specialty followed by palliative care and then oncology/hematology (Table 1).

**Table 1:** Characteristics of participants (N = 14).

		N
**Gender**	Male	7
	Female	7
**Provider Type**	Resident, MD/DO	5
	Attending, MD/DO	7
	Nurse Practitioner	2
**Specialty**	Oncology	2
	Palliative Medicine	4
	Internal Medicine	7
	Family Medicine	1
**Type of Practice**	Primarily out-patient	5
	Primarily in-patient	9

Three related challenges leading to misclassification on death certificates were identified: (1) infrastructural and procedural challenges encountered when completing death certificates; (2) clinical uncertainty and the approaches providers take to determine the cause of death; and (3) unique challenges posed by decedents with a recent or remote history of cancer.

### Infrastructural and procedural challenges

**Fig 1:**
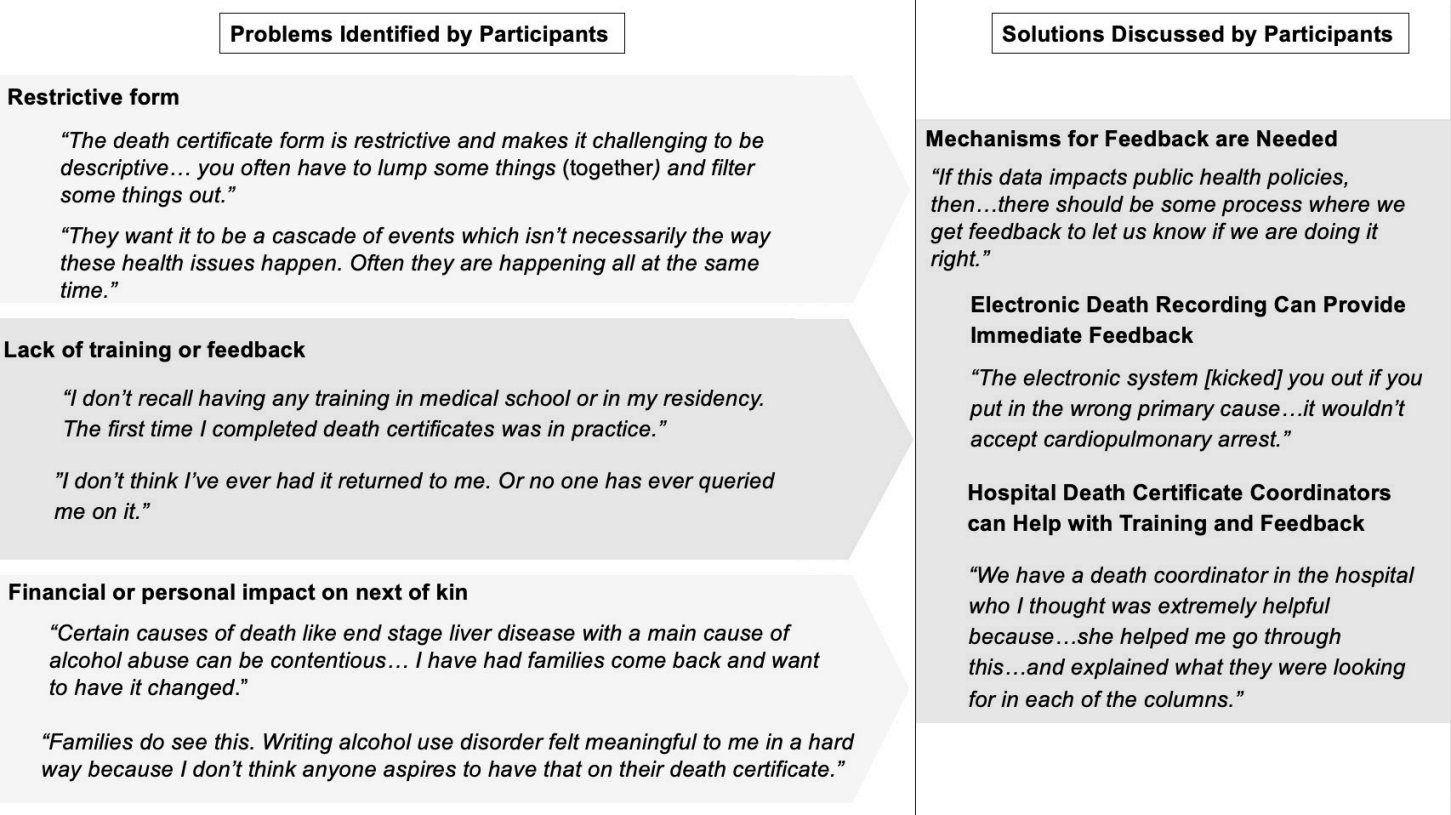
Factors that can introduce error in death certificates and solutions discussed by participants.

Participants described several factors relating to the infrastructure and procedure involved in death certification; the structure of the form and its impact on data that can be entered, the lack of training and feedback, and the influence of the next of kin (Fig 1).

A major challenge described in all the focus groups was the restrictiveness of the death certificate form whose rigid structure may force providers to alter the sequence of events and omit important information as they describe the cause of death. Another commonly discussed challenge was the lack of standardized training or formal feedback for death certificates. Ten of 14 providers reported not having received any training on death certification in medical school or residency and five did not complete their first death certificate until after residency. Only two of 14 providers reported receiving formal feedback from state or hospital organizations on the quality of their death certificates. An additional topic that was discussed in a smaller number of focus groups was the impact of the next of kin on the information included in the death certificate. Although providers did not report changing the death certificate based on family wishes, they did note the challenging circumstances that arose when factors such as life insurance, military exposures to carcinogenic agents and disease stigma were raised during the death certification process because of perceived financial or personal implications for the next of kin.

Providers identified several potential solutions to the infrastructural and procedural challenges that were raised during the focus groups. Most targeted the lack of training or feedback, with several providers emphasizing that both training and consistent feedback are necessary for a document that is widely used for research and public health. Providers supported two main solutions: electronic death certification, and access to hospital death coordinators for deaths occurring in the hospital. Electronic death certification is used in several states already and was seen by many providers as a way to build instantaneous feedback into the process. Some providers described electronic systems in other states that would reject invalid diagnoses, such as mechanisms of death, and would catch errors such as fields left blank or information entered in the wrong area. Providers working in the inpatient setting described the benefit of a hospital death coordinator for proving feedback and ongoing training. Death coordinators use their expertise in certification to screen for errors and provide real-time feedback and training to staff when they encounter challenges.

### Clinical uncertainty and approaches to determining the cause of death

Clinical uncertainty, described in many situations, posed a significant challenge for providers. Two main scenarios led to clinical uncertainty for both community and hospitalist providers; unexpected deaths and deaths following a prolonged period without medical care. Both of these challenges were present in the case study presented during the focus group (S2 Appendix) and resulted in a wide variety of causes of death reported by the providers at the conclusion of the case study (Table 2).

**Table 2:** Cause of death selected for the case study.

Immediate Cause of Death	Responses
Cardiac arrest	3
Cardiovascular disease	2
Colon cancer	2
Cerebrovascular disease	2
Respiratory failure	1
Cannot determine–would request autopsy	3
Cannot determine–no cause of death provided	1

**Fig 2:**
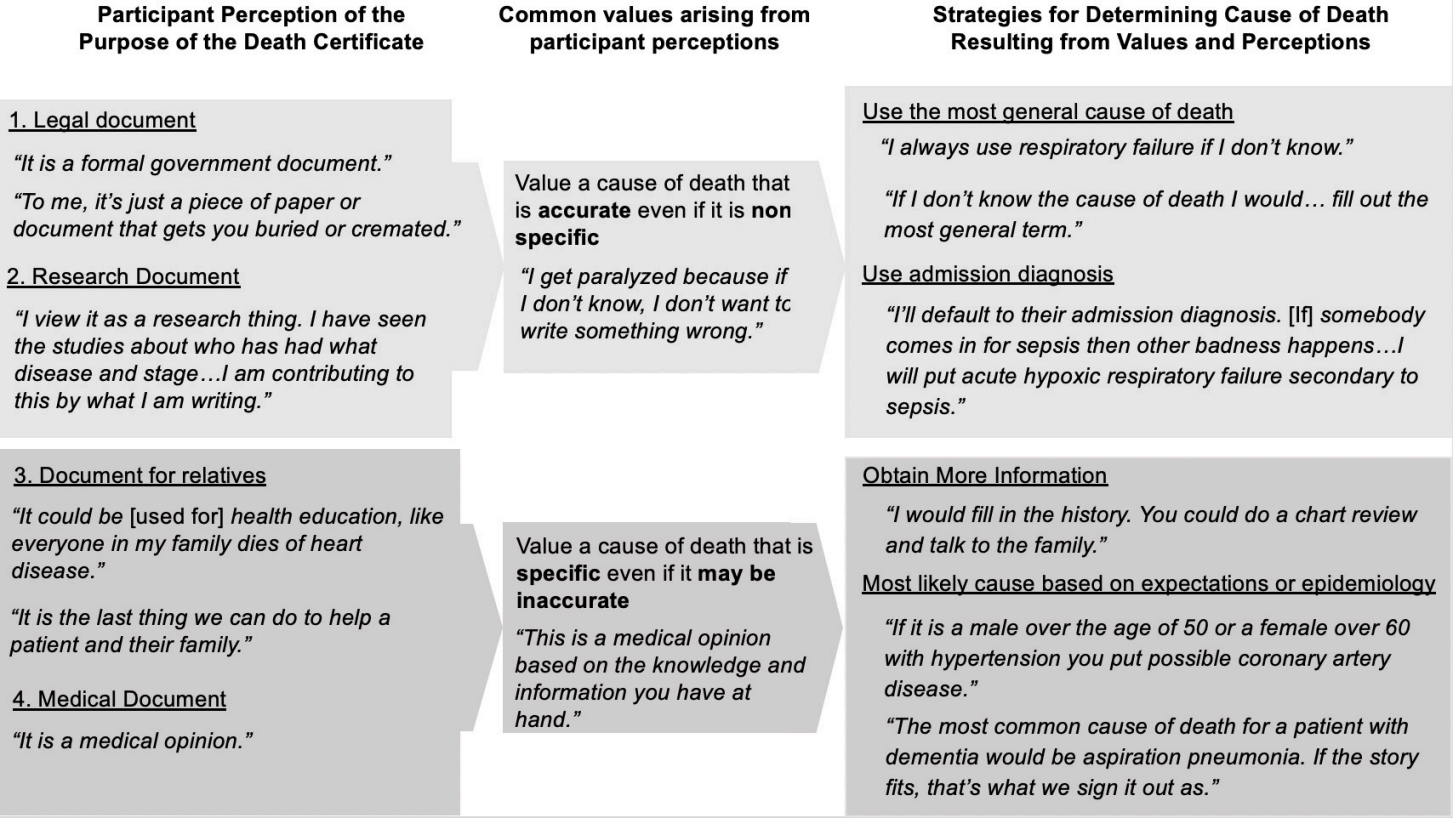
Relationship between participant perception of the purpose of death certificates and their strategies for determining cause of death when faced with clinical uncertainty.

Providers reported two main approaches to determining the cause of death in situations of clinical uncertainty (Fig 2), depending on their perceptions of the purpose of death certificates. Eight of 14 providers viewed death certificates as being legal documents or data for research and tended to value accuracy over specificity, listing the broadest cause of death that is accurate, even if it is a mechanism of death such as cardiac arrest, or listing the admission diagnosis if the patient dies while in the hospital. In contrast, six of 14 providers viewed the death certificate as a document intended for the medical record or for use by families and they tended to value a more specific diagnosis, even if it may be inaccurate. These providers described a strategy of making an educated guess on the cause of death based on a wide variety of tools including the medical record, conversations with family members and demographic information.

### Patients with cancer

**Fig 3:**
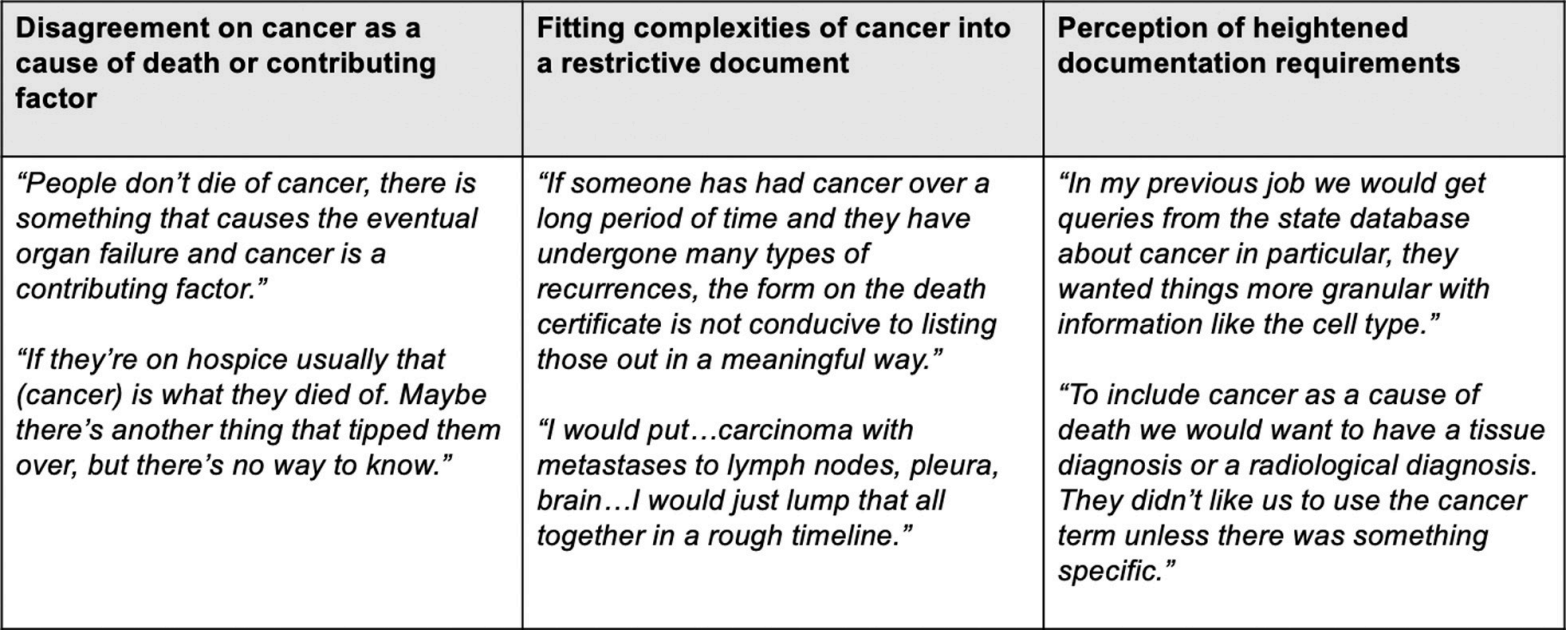
Challenges encountered when documenting cancer on the death certificate.

Three specific challenges were identified in completing death certificates for patients with cancer (Fig 3). One was deciding if cancer should be listed as the immediate or underlying cause of death. Some providers believed that if a patient died from an aggressive cancer, the cancer should be listed as the immediate cause of death. Other providers had been taught that cancer should not be listed as the immediate cause of death. The second challenge was fitting the complexities of the diagnosis or patient history into the death certificate in a meaningful way. Many providers described patients with long histories of cancer with periods of remission and multiple recurrences which did not fit easily into the confines of the death certificate. The third challenge reported by providers was the heightened documentation requirements that some providers perceived exist for cancer. Many felt that exact tissue diagnosis, location of the primary tumor and sequence of metastatic spread should be included in the death certificate. Some oncologists and palliative care providers also included pathology information and a list of remissions and recurrences when applicable. This degree of detail was challenging for many of the hospitalist and community providers that did not have extensive records of patients’ cancer history. Five providers reported receiving queries from the state cancer registry regarding death certificates listing cancer as a cause of death. Because of this impression of heightened documentation requirements, many participants felt that cancer should not be included on a death certificate unless there was clinical certainty.

## Discussion

This study identified three main types of challenges encountered by providers when completing death certificates; infrastructural and procedural challenges, clinical uncertainty and the unique challenges posed by patients with cancer. Focus group participants tied these challenges to potential sources of error in death certificates, both directly and indirectly.

An overarching challenge identified by providers was a lack of formal training or feedback about a certification process that is often inflexible and, with the exception of new electronic systems, seldom provides real-time feedback or training to optimize the data. Focus group participants agreed that the certification process can lead to inconsistency and error, and it has long been recognized that these ultimately contribute to unreliable public health data [18–22]. Several of the quotations from our focus group discussions illustrate common areas of confusion in the certification process including the use of mechanisms of death, uncertainty around how to handle cancer, and when to refer for an autopsy. All of these areas of confusion and consequent errors in death certification could be addressed by uniform, standardized training and ongoing feedback.

There were also several potential sources of error uncovered in conversations around how providers determined the cause of death. Providers that valued the accuracy of the death certificate were careful to only document diseases, conditions or processes that they could verify; this approach was problematic when there was clinical uncertainty around the cause of death. It was concerning that physicians’ views of the role and importance of death certificates to public health and to families might affect the approach they took to select a cause of death. In addition, several participants reported that, when unable to make a definitive diagnosis, they would list mechanisms of death such as cardiac or respiratory arrest; however, these mechanisms simply attest to the fact of death and they do not describe its etiology. If used at all, mechanisms of death must always be accompanied by cause of death: “the disease, abnormality, injury, or poisoning that caused the death” [27]. When the cause of death was not definitively diagnosed, it is acceptable to list it as “probable” or “likely”. Contrary to the understanding of some focus group participants, it is also acceptable to list “old age” as a cause of death in appropriate circumstances [28].

Providers faced with clinical uncertainty about the cause of death described how they combined clinical judgement with their expectations based on demographics and epidemiological trends; they acknowledged that this approach was a potential source of misclassification in the cause of death. Several group members noted that cardiovascular causes of death are often chosen because of expectations based on demographics. For example, one participant mentioned being asked to follow an algorithm for unwitnessed or sudden events that would attribute deaths to cardiovascular disease in all males over age 50 and to females over age 60 with a history of hypertension. In a concerning form of circular logic, this approach is likely to perpetuate and amplify inaccuracies in the data, exaggerating the importance of one cause of death over others and potentially obscuring important trends. This finding is supported by several studies that have demonstrated that death certificates substantially overestimate cardiovascular mortality [21, 29, 30]. Since these data are important to track diseases over time, determine research and funding priorities and inform new clinical guidelines, death certification data must be accurate and trustworthy.

Our study highlighted uncertainty over how to document cancer on the death certificate. In the hypothetical and intentionally vague case study, participants took varied approaches when the role of cancer was uncertain and the death was unwitnessed. Some participants described cancer as the immediate cause of death while others described it as the underlying cause of death and several participants omitted it completely. One focus group discussion centered around the idea that people don’t die of cancer, but of something else (e.g. anorexia-cachexia, infection) with cancer as a contributing factor. However, when cancer causes death through such a process, it should be listed as a proximate cause of death and not a contributing cause [27]. Many participants believed that in order to include cancer on the death certificate, a tissue biopsy was necessary. Ideally, a death certificate that indicates neoplasm as a cause of death should include the primary site, behavior (benign/malignant), cell type, grade, and the part or lobe of organ affected [27]. But again, a “probable” or “likely” diagnosis is also acceptable when such data are not available. Our results also highlighted variability in distinguishing deaths “from cancer” and deaths “with cancer” and raise concerns about the reliability of national cancer mortality statistics [18, 22, 31].

A 2005 focus group study by McAllum et al. examined the completion of death certificates by general practitioners in New Zealand [32]. Consistent with our findings, they demonstrated that clinical uncertainty posed a significant challenge for providers in the completion of death certificates. Specific challenges were related to gaps in care and sudden deaths, and there was a tendency to fall back on a diagnosis of cardiovascular disease or myocardial infarction as the cause of death when faced with clinical uncertainty. A second theme was the important role of the deceased’s family in determining the cause of death; this was a less prominent issue in our study although some providers described anger from family members over the certification of stigmatizing conditions such as alcoholism. McAllum et al. reported the same circular logic seen in our study whereby providers who do not know the true cause of death may assign one based on their expectations according to patient characteristics such as age, gender, and ethnic group. Uncertainty is likely when physicians are asked to certify deaths of patients they have not treated, or if the patient has not recently sought medical attention. In recent decades, and particularly since the Joint Commission removed the requirement of a 20% autopsy rate by hospitals, there has been a dramatic decline in autopsy rates to only 8.5% in 2007 [33]. “Gold standard” data from autopsies would help stop this cycle of uncertainty and allow death certificates to more accurately reflect important trends in population mortality [14, 34, 35].

Our findings highlight the need for improvement of specific aspects of the death certification process. Several studies have shown significant reductions in errors with standardized training programs, especially interactive programs that are periodically reinforced [36, 37]. For example, a training program in Spain reduced the error rate among a group of residents from 71% to 9% [37]. The Centers for Disease Control [27], World Health Organization [38], and others have established educational materials on death certificates that can be used to build a structured training program for all providers that are eligible to complete death certificates. A mechanism for structured, timely feedback has also been shown to reduce error in death certificates [39].

One of the quickest and least resource intensive methods of providing feedback is through an electronic death reporting system (EDRS), such as the New Hampshire Electronic Cause of Death mobile application [40]. Unlike paper forms, electronic systems can be structured to provide real-time feedback, for example by rejecting mechanism of death such as cardiac arrest, and by offering “help” menus to assist in areas of doubt [2]. Feedback is particularly important during public health crises such as the opioid epidemic and Covid-19 pandemic to ensure that deaths are certified using consistent methods, and that timely, accurate data are made available for public health officials and policy makers to monitor the crisis and its management. However, electronic certification systems cannot be expected to overcome errors relating to poor training and uncertainty over causes of death, and the use of dropdown menus and restrictions within the software may even introduce new errors and biases. Variation in software and in the time at which states and territories have adopted electronic certification technology [41] may also impair the interpretation of trends in causes of death. These limitations must be studied within and between states that use different EDRS software to quantify the impacts of “old” and “new” biases on misclassification of causes of death, but without autopsy confirmation this task may be difficult.

A significant strength of this study was the variety of providers that participated, including physicians and nurse practitioners from both in-patient and outpatient settings with backgrounds in four specialties and a wide variety of experience levels including residents and attendings. Another strength was the ability to conduct multiple focus groups to the point of redundancy which reduced the likelihood of missing major themes. Limitations of the study included its focus on two health systems in New England, potentially limiting the generalizability of these findings to other U.S. settings. The relatively small size of some of the focus groups and greater proportions of hospital-based participants may have limited the extent of novel ideas and quality of group discussion, and the use of focus groups rather than semi-structured interviews may have introduced group biases.

We propose the following strategies to improve the accuracy of death certificates. (1) Expand the use of electronic death certification software to provide guidance, validation and feedback in real time to improve incoming data. It will be critical to design and assess such software carefully to prevent the introduction of new biases. Immediate feedback via prompts within the software would promote the collection of more accurate data on specific diseases such as cancer and heart disease, including whether these diseases caused, contributed to, or were not relevant to the death. (2) Train all physicians and associate providers in the completion of death certificates both before graduation and as part of continuing education. (3) Change sudden death classification to include a place to record whether the death was witnessed or unwitnessed to provide information relating to certainty of the diagnosis. (4) Reinstate autopsy requirements in order to validate causes of death when diagnoses are uncertain in order to improve confidence in mortality statistics.

In conclusion, focus group discussion highlighted substantial challenges and potential sources of error in the death certification process, and provided a map for improvement to this crucial piece of information that we use to make large scale policy and public health decisions. It is critically important that the medical community recognize the impact of inaccurate death certification on population statistics and act to address it.

## Supporting information

S1 Appendix Questions used in the focus groups. (DOCX)

S2 Appendix Case study. (DOCX)

S1 File (DOCX)
