# Supplementary material for: Provider reported challenges with completing death certificates: A focus group study demonstrating potential sources of error
Source: PLoS One. 2022 May 20;17(5):e0268566. doi: 10.1371/journal.pone.0268566 (PMC9122187; doi:10.1371/journal.pone.0268566)
Supplement: S1 Appendix — (DOCX) [file pone.0268566.s001.docx]

S1 Appendix: Questions Used in the Focus Groups

| 1. When and how did you learn to fill in the cause of death on death certificates? What factors do you consider? |
| --- |
| 2. How do you decide on the immediate cause of death, underlying causes of death, and contributory illness? |
| 3. How did you determine the cause of death in the case study? In your normal practice, how do you determine the cause of death when you are uncertain? |
| 4. When and how do you include cancer on a death certificate? |
| 5. What do you do when you do not know the exact cause of death and how do you decide whether to refer to the medical examiner?  [If not discussed, Do race, age, ethnicity or gender affect what you report as the cause of death when you are uncertain?] |
| 6. Are there any aspects of the death certificate process that you are uncomfortable with or that frustrate you? |
| 7. Are you ever asked to complete a death certificate for the patient of a colleague, and if you have no personal knowledge of the patient, how do you deal with those requests? |
| 8. Is there anything else you would like to discuss regarding your experiences with death certificates? |
