# Supplementary material for: Provider reported challenges with completing death certificates: A focus group study demonstrating potential sources of error
Source: PLoS One. 2022 May 20;17(5):e0268566. doi: 10.1371/journal.pone.0268566 (PMC9122187; doi:10.1371/journal.pone.0268566)
Supplement: S2 Appendix — (DOCX) [file pone.0268566.s002.docx]

S2 Appendix: Case Study

Mrs. Williams is an 82-year-old female that has been a patient in your practice for 20 years. Her husband died six months ago from Parkinson’s disease. For the year prior to his death Mrs. Williams was her husband’s primary caregiver causing her to neglect her own health. Her medical history consists of hypertension, hypercholesterolemia, transient ischemic attack (TIA) 1 year ago, and stage III colon cancer 2 years ago which was treated with surgery and adjuvant chemotherapy.

Mrs. Williams was found unresponsive in bed this morning by a neighbor. The neighbor noted that in the past 2 months Mrs. Williams had lost a lot of weight. Her daughter lives in Canada and refuses permission for an autopsy.
