# Supplementary material for: Provider reported challenges with completing death certificates: A focus group study demonstrating potential sources of error
Source: PLoS One. 2022 May 20;17(5):e0268566. doi: 10.1371/journal.pone.0268566 (PMC9122187; doi:10.1371/journal.pone.0268566)
Supplement: S1 File — (DOCX) [file pone.0268566.s003.docx]

**Problems Identified by Participants:**

*“The death certificate form is restrictive and makes it challenging to be descriptive… you often have to lump some things* (together*) and filter some things out.”*

*“They want it to be a cascade of events which isn’t necessarily the way these health issues happen. Often they are happening all at the same time.”*

*“I don’t recall having any training in medical school or in my residency. The first time I completed death certificates was in practice.”*

*”I don’t think I’ve ever had it returned to me. Or no one has ever queried*

*me on it.”*

*“Certain causes of death like end stage liver disease with a main cause of alcohol abuse can be contentious… I have had families come back and want to have it changed*.”

*“Families do see this. Writing alcohol use disorder felt meaningful to me in a hard way because I don’t think anyone aspires to have that on their death certificate.”*

**Solutions Discussed by Participants**

*“If this data impacts public health policies, then…there should be some process where we get feedback to let us know if we are doing it right.”*

*“The electronic system [kicked] you out if you put in the wrong primary cause…it wouldn’t accept cardiopulmonary arrest.”*

*“We have a death coordinator in the hospital who I thought was extremely helpful because…she helped me go through this…and explained what they were looking for in each of the columns.”*

**Participant Perception of the Purpose of the Death Certificate**

*“It is a formal government document.”*

*“To me, it’s just a piece of paper or document that gets you buried or cremated.”*

*“I view it as a research thing. I have seen the studies about who has had what disease and stage…I am contributing to this by what I am writing.”*

*“It could be [used for] health education, like everyone in my family dies of heart disease.”*

*“It is the last thing we can do to help a patient and their family.”*

*“It is a medical opinion.”*

**Common values arising from participant perceptions**

*“I get paralyzed because if I don’t know, I don’t want to write something wrong.”*

*“This is a medical opinion based on the knowledge and information you have at hand.”*

**Strategies for Determining Cause of Death Resulting from Values and Perceptions**

*“I always use respiratory failure if I don’t know.”*

*“If I don’t know the cause of death I would… fill out the most general term.”*

*“I’ll default to their admission diagnosis.* [If] *somebody comes in for sepsis then other badness happens…I will put acute hypoxic respiratory failure secondary to sepsis.”*

*“I would fill in the history. You could do a chart review and talk to the family.”*

*“If it is a male over the age of 50 or a female over 60 with hypertension you put possible coronary artery disease.”*

*“The most common cause of death for a patient with dementia would be aspiration pneumonia. If the story fits, that’s what we sign it out as.”*

**Challenges Encountered when Documenting Cancer on the Death Certificate**

*“People don’t die of cancer, there is something that causes the eventual organ failure and cancer is a contributing factor.”*

*“If they’re on hospice usually that (cancer) is what they died of. Maybe there’s another thing that tipped them over, but there’s no way to know.”*

*“If someone has had cancer over a long period of time and they have undergone many types of recurrences, the form on the death certificate is not conducive to listing those out in a meaningful way.”*

*“I would put…carcinoma with metastases to lymph nodes, pleura, brain…I would just lump that all together in a rough timeline.”*

*“In my previous job we would get queries from the state database about cancer in particular, they wanted things more granular with information like the cell type.”*

*“To include cancer as a cause of death we would want to have a tissue diagnosis or a radiological diagnosis. They didn’t like us to use the cancer term unless there was something specific.”*

**Causes of Clinical Uncertainty**

*“When they’ve only been here for a few hours and they pass away quickly and you really don’t have any other information, you have no idea what the actual cause of death is.”*

*“Sometimes it’s ambiguous because the patient decided to go CMO (*comfort measures only) *before you could figure out the cause of death.”*

*“But we had no past, like he never saw a doctor for the past twenty years. We had no idea what their past medical treatment would be.”*

*“There are a lot scenarios where you don’t have a diagnosis, they’re not being monitored, or scanned and they pass away pretty quickly.”*
